# More Colors in a Rainbow

**DOI:** 10.1007/s10508-015-0627-9

**Published:** 2015-09-25

**Authors:** Ine Vanwesenbeeck

**Affiliations:** Rutgers, POB 9022, 3506 GA Utrecht, The Netherlands; Department of Interdisciplinary Social Sciences, Utrecht University, PO Box 80140, 3508 TC Utrecht, The Netherlands

With her sexual configurations theory (SCT), van Anders (2015) wants to solve a number of problems with the current conceptualization of sexual orientation as defined by the sex of the partner(s) one is attracted to and habitually indicated with the Kinsey score varying from completely heterosexual to completely homosexual.

I agree with van Anders that the conventional conceptualization of sexual orientation has a number of shortcomings. Indeed, why would the sex of our desired partners be the prime organizing feature of our sexuality? What is the role of partners’ genderedness or of other major person characteristics? “Biological sex” is not as clear-cut a category to justify its monopoly position in sexual orientation theory in the first place. Obviously, reliance on (difference or similarity between) discrete binary categories ill-caters for those who do not fit them, such as intersex and trans people. Moreover, Kinsey scores are extremely crude. And they are ill-catering for asexuality and inconsiderate of solo sexual experiences. Besides, “the score” assumes stability where we know dynamic fluidity exists. And indeed, the empirical differences in scores on the various domains of sexual orientation (attraction, self-identification, and behavior) add to the confusion of what we are exactly referring to when talking about “sexual orientation.”

Because of all these problems, van Anders proposes SCT, aimed to better “address the complexities of actual people’s sexualities” and she wants to do so in an inclusive, norm free, and non-discriminatory way. She claims SCTs usefulness for self-knowledge and sexual minorities’ empowerment, as well as for neuroendocrinological, social, and health-related research methodologies and clinical practice. I gladly take the opportunity to share why I feel she has come a long way in reaching those goals while at the same time not fully fulfilling her ambitious promises. First, a short description of the SCT is in order.

## A Quick Tour of SCT as I See It

The key premise of the SCT is that sexual orientation diversity is fundamentally multidimensional. Gender, partner number, and the eroticism/nurturance distinction are added to “biological sex” as prime dimensions of one’s sexual preference. The parameter eroticism/nurturance mixes with partners’ gender/sex and partner number to allow for variability of combinations. In addition, the sexual configuration may comprise a (any?) further number of parameters (“parameter n”) such as age of the preferred partner(s), preference for consent, and/or physical violence and/or forms of kink.

The assembly or notation of one’s sexual configuration implies scores on each dimension or parameter. The total mapping of scores on the various parameters is one’s configuration. There are two main strands in one’s sexual configuration, one relating to partnered and one to solitary sexuality. Unfortunately, van Anders limits herself to the first strand only in her article. Parameters gender/sex, partner number, and eroticism/nurturance are most comprehensively described.

The parameter gender/sex deals with someone’s orientation to male and/or female sexes and/or genders. In contrast to Kinsey’s one-dimensional line, van Anders introduces a widening/narrowing spiraling circle. Actually, she introduces three of them: one for attraction guided by partners’ sex, one by partners’ gender, and one by a combination of both. On each of these spiraling circles, attraction (or behavior/status) can vary in terms of binarity, specificity, and strength. A non-binary gender/sex attraction concerns, for example, people who challenge the gender/sex binary or exist outside of it (e.g., intersex identified individuals). Together with attraction to cis men or women and/or traditional femininity/masculinity, these options are positioned at the specific side of the circle. Non-specifically, “pan”-oriented individuals lean towards any gender and/or sex. When one’s gender/sex orientation is less strong (to be notated where the spiral narrows), partners’ gender/sex is of less importance compared to, for instance, other characteristics such as power, humor, servitude, just to name a few (physical appearances are remarkably absent in van Anders’ lay-out).

The parameter partner number deals with preference for (or sexual practice with) zero, one, two, or more partners. This parameter allows for asexual, monogamous, bisexual, and polyamorous orientations (or statuses). Again, the possible variation in terms of binarity, specificity, and strength can be scored. A binary orientation refers to a distinct preference for either one or more partners while someone with a non-specific orientation could not care less about their number. Finally, orientations can vary according to preference for eroticism versus nurturance, a distinction which may again differ in terms of binarity, specificity, and strength. When multiply oriented, one can be erotically interested in some but nurturantly in other partner(s). Whereas the concept sociosexuality refers to a general liking of having erotic sex with multiple partners, the SCT permits all possible combinations. The SCT is also sensitive to, for instance, “Gray-A” by allowing for scoring the strength and thus degree of asexuality instead of assuming a distinct either-or.

Scores on all these dimensions together form one’s sexual configuration, which is a complex of different, interconnected but distinct orientations, of “multiple multifaceted facets” of one’s sexuality. A sexual configuration is clearly much more than a numbered dot on a line. It is a multidimensional conceptual space squiggly marked out between assembled scores on the various parameters that I visually imagine standing there like spiraling cones in what van Anders calls the Sexual Configuration Landscape, which I picture like an undulating blanket tied up between a potentially infinite number of parameter stalagmites.

## A Laudable Exercise!

First and foremost, I feel van Anders must be lauded for a clever, creative, geometrically cunning, and inspiring intellectual exercise. She has no doubt managed to tackle some of the problems with conventional conceptualizations of sexual orientation. Notably, the introduction of non-binary positions and attractions makes the theory fully useable for and inclusive of categories of “real people” otherwise left out and considered difficult to locate. Granting gender a conceptual space next to biological sex has done away with objectionable bio-essentialism. And the introduction of multiple parameters in combination with the possibility to vary on them in terms of specificity and strength has hugely expanded the possible number and subtleties of orientations and given enormous depth to our thinking about diversity. With its openness also to punishable, paraphilic, and “grey” attractions, it is inclusive beyond what any theory on sexual diversity that I am aware of has ever achieved. The well-sharpened, highly sensitive diversity glasses that van Anders puts on are undeniably conducive to doing justice to the complexity and diversity of our lived realities.

As such, the attentive value of the SCT is high. In accordance with van Anders’ claim, I can see its usefulness as a tool for self-investigation and self-knowledge. I can see its emancipatory value as well as its clinical importance. I tend to see limitations to its empirical usefulness, however.

## With Limitations

I am not fully convinced by how van Anders envisions the ways in which the SCT can make the complexity and diversity of our lived realities empirically visible. Clearly, qualitative descriptions, in *N* = 1 or *N* > 1 studies, may provide theoretical and empirical insights and may certainly have the attentive, awareness raising, and emancipatory value already referred to. And maybe some quantitative measurements may provide individual and group scores on separate aspects of (separate parameters of) sexual configurations. But I am lost as soon as I try to think about overall classifications of sexual configurations. The complexity and uniqueness of each configuration simply forbids it. “Not all configurations are as complex,” van Anders puts forward and, of course, she is right. In quite a number of configurations, dimensions may sooner coincide than branch off; the possible complexity and subtlety is wasted on those. But I have difficulty seeing how configurations that fully exploit the possibilities of being multifaceted and detailed can yet again be classified in meaningful categories of sexual identities. van Anders describes a number of identities in SCT terms. She notes, for instance, that specific configurations in a specific context and in intersectional relation to other personal factors may be experienced as identities, such as wink, bear, or stud. Indeed, those and many other identities may be well characterized in SCT terminology. But as soon as they are being used as a category of configurations, as a classification, they detract from what I feel is SCT’s main strength and gain, namely its “let a thousand flowers bloom” principle, its pontifical opening of a wide-ranging fan of possible configurations and identities based on multiple descriptive dimensions. What the SCT, in my opinion, shows best are the many nuanced colors in the rainbow; reduction to a couple of primary ones is a step backwards.

The fact remains that the relation between one’s assembled configuration and one’s lived identity is not straightforward. Every dimension added to the SCT, or every parameter stalagmite added to the configuration landscape, makes it harder to identify the landscape as a singular identity, a coherently, classifiable lived experience. Moreover, as was the case with thinking in terms of a singular orientation, the distinction and possible deviation between attractions (or orientations), behaviors (or statuses), and self-identifications remains an issue. van Anders proposes different notations when demarcating the different aspects in one’s Sexual Configuration Landscape, like a-dots for attractions and s-dots for statuses. Well, that does not make me too happy.

Other problems that the SCT aimed to solve were not essentially solved either. Although solitary sexuality has been given principle space in the sexual configuration, it remains distinct from partnered sexuality and the ways solitary sexuality may affect one’s overall sexual configuration remain largely unexplored. Likewise, van Anders acknowledges the possible temporality and fluidity of sexual configurations, but does not do much more to incorporate that notion in one’s overall configuration than was done in singular orientation theory. Luckily, some problems remain to be solved.

## The Future of Sexual Configurations

With van Anders’ introduction of SCT, our thinking about sexual diversity has gained substantial and irreversible progress. SCT offers a sophisticated tool that is implementable towards increasing understanding, normalization, communication, and capacity building in relation to sexual orientation/configuration. SCT provides an open and future-proof vision adaptable to attractions lived by new generations beyond our imagination right now. What we now need to conceptualize and investigate are the conditions under which such a colorful configurations diversity may also be societally supported. What are the consequences for legal and educational systems? What does it mean for sexual minorities’ self-organization and emancipatory movements? How to find a proper balance between specificities and generalities in health care and social work? How to counter conservative or even reactionary powers stepping up their efforts to reduce our intimate lives to the monogamous heterosexual variant? Although important, such questions do not refrain from the relevance of the leap forward van Anders has presented us with. Let us start implementing!
